# The Elevated Secreted Immunoglobulin D Enhanced the Activation of Peripheral Blood Mononuclear Cells in Rheumatoid Arthritis

**DOI:** 10.1371/journal.pone.0147788

**Published:** 2016-01-27

**Authors:** Yujing Wu, Wensheng Chen, Hengshi Chen, Lingling Zhang, Yan Chang, Shangxue Yan, Xing Dai, Yang Ma, Qiong Huang, Wei Wei

**Affiliations:** Institute of Clinical Pharmacology, Anhui Medical University, Key Laboratory of Anti-inflammatory and Immune Medicine, Ministry of Education, Anhui Collaborative Innovation Center of Anti-inflammatory and Immune Medicine, Hefei, 230032, China; Yale University, UNITED STATES

## Abstract

Immunoglobulin D (IgD) is a surface immunoglobulin that is expressed as either membrane IgD (mIgD) or secreted IgD (sIgD). Researchers have shown that sIgD is often elevated in patients with autoimmune diseases. The possible roles of sIgD on the function of peripheral blood mononuclear cells (PBMCs) in rheumatoid arthritis (RA) are still unclear. In this study, we compared the expression of sIgD, mIgD and IgD receptor (IgDR) in RA patients and healthy controls, and investigated the effect of sIgD on the function of PBMCs. We found that the levels of sIgD, mIgD and IgDR were significantly higher in RA patients compared with healthy controls. The concentrations of sIgD were positively correlated with soluble receptor activator of nuclear factor-κB ligand (sRANKL), rheumatoid factor (RF) and C-reactive protein (CRP) in RA patients. Strikingly, IgD could enhance the proliferation of PBMCs and induce IL-1α, IL-1β, TNF-α, IL-6 and IL-10 production from PBMCs. Moreover, the percentage of activated T cell subsets (CD4^+^CD69^+^, CD4^+^CD154^+^) and activated B cell subsets (CD19^+^CD23^+^, CD19^+^CD21^+^, CD19^+^IgD^+^ and CD19^-^CD138^+^) were increased by IgD. The percentage of unactivated T cell subset (CD4^+^CD62L^+^) and immature B cell subset (CD19^+^IgM^+^IgD^-^) were decreased by IgD in PBMCs. Furthermore, the expressions of IgDR on T and B cells were significantly increased by treatment with IgD. Our results demonstrate that IgD enhanced the activation of PBMCs, which may contribute to RA pathogenesis. Therefore, IgD could be a potential novel immunotherapeutic target for the management of RA.

## Introduction

Immunoglobulin D (IgD) is an immunoglobulin (Ig) isotype that can be expressed as membrane IgD (mIgD) or secreted IgD (sIgD). IgD accounts for less than 1% of Igs in blood [1–3], suggesting that it forms a minor component of serum. As an important marker of B cell development and maturation, mIgD is co-expressed with IgM on more than 90% of mature B cells [4, 5]. IgD promotes immune defense which cause inflammation and tissue damage by inducing the activation and infiltration of immune cells [6, 7]. Like other Ig isotypes, IgD also has a specific Fc receptor (IgDR). The existence of functional Fc receptors for IgD on mice and human T cells has been reported [8–10]. However, the functional and molecular characteristics of IgD and IgDR still remain elusive.

Accumulating evidences have suggested that IgD may contribute to disease pathogenesis. For example, IgD-producing B cells are elevated in systemic immune system in patients with hyper-IgD syndrome (HIDS) [11]. sIgD levels are increased in autoimmune diseases such as rheumatoid arthritis (RA), systemic lupus erythematosus (SLE), Sjogren’s syndrome and autoimmune thyroiditis [12–13]. High expression of sIgD was found to be related to high levels of protein-like sediments and cell necrosis in kidney, spleen and liver in transgenic mice [14]. IgD-secreting plasmacytomas in mice generate augmented primary and secondary humoral immune responses after antigen challenge [15]. Similar to our previous findings showing that IgD could induce human Burkitt lymphoma Daudi cell proliferation by accelerated G1/S transition [16], we propose that, abnormal sIgD levels might cause imbalance immune system, which play an important role in autoimmune diseases such as RA.

RA is a chronic systemic inflammatory disease characterized by inflammation of the joint synovial tissue. In recent years highly selective immunologic therapies have been developed. For B cell depletion therapy in RA, rituximab (anti-CD20 monoclonal antibody) has been proven effective for reducing the clinical signs and symptoms of RA [17]. However, rituximab, non-selectively depletion of B cell, may lead to disorders of the immune system that can break autoimmune homeostasis. Lately, Nguyen TG reported that anti-IgD treatment selectively depletes mature B cells in collagen-induced arthritis (CIA) mouse model, which strongly suggests that IgD may provide a new therapeutic target for B cell regulation in autoimmune diseases [7].

It is unclear how sIgD and IgDR are expressed in RA, and the possible role of sIgD on the function of peripheral blood mononuclear cells (PBMCs) in RA pathogenesis. Therefore, in this study, we compared the expression of sIgD, mIgD and IgDR in RA patients and healthy controls, and subsequently investigated the effect of sIgD on the function of PBMCs. The results showed that the expression of IgD and IgDR in RA patients were significantly higher than those in healthy controls. The concentrations of sIgD were positively correlated with soluble receptor activator of nuclear factor-κB ligand (sRANKL), rheumatoid factor (RF) and C-reactive protein (CRP) in RA patients. Furthermore, IgD could enhance the proliferation of PBMCs, induce the production of cytokines, and activate T and B cells and simultaneously promoted the expression of IgDR, which may contribute to RA pathogenesis.

## Materials and Methods

### Patients

The study protocol was carried out in accordance with the Declaration of Helsinki (2008) and approved by the Ethics Committee of Anhui Medical University. Written informed consent was obtained from each participant before the start of the study. Patients eligible for this study included adults (aged 18–80 years) with RA according to the revised 1987 American Rheumatism Association criteria [18] for < 6 months, and who had active disease despite methotrexate (MTX), disease-modifying anti-rheumatic drug (DMARD), and/or glucocorticoid therapy at the time of screening. Active RA was defined as persistent disease activity with ≥ 6 swollen and ≥ 6 tender joints, as screening baseline. Consequently, patients who have been treated with MTX, DMARD, glucocorticoid, or any combination there of for ≥ 6 months prior to screening were excluded. 54 RA patients at the department of rheumatology and immune (The First Affiliated Hospital of Anhui Medical University, Hefei, Anhui, China) were enrolled, as well as 42 aged matched healthy donors. All donors were evaluated through medical histories, physical examinations, and routine clinical laboratory tests.

### Clinical and Serological Assessments

History, baseline characteristics and demographic data were recorded and physical examination was performed in each patient at the beginning of the study. The levels of sIgD, human sRANKL, anti-cyclic citrullinated peptide (anti-CCP), CRP were determined in serum samples using the ELISA method (Abcam, USA) according to the manufacturer's instructions. RF was determined by quantitative nephelometry using rheumatoid factors test kit (Kang Hua life science, China). Erythrocytes sedimentation rate (ESR) was tested by Westergren method.

### Isolation of PBMCs

PBMCs were isolated from peripheral blood of donors by ficoll gradient centrifugation and re-suspended in 1.5 ml PBS and 1% BSA (1 × 10^6^ cells/100 μl). Cells were not used if viability did not exceed 80%. To obtain peripheral blood leukocytes, monocytes were depleted from PBMCs via their adhering to plastic culture flasks.

### The Expression of mIgD and IgDR Were Measured by Flow Cytometry Analysis

PBMCs collected from RA patients and healthy controls were incubated with various combinations of monoclonal antibodies (mAbs) for 30 min at room temperature. The total T cells (CD3^+^), helper T cells (CD3^+^CD4^+^), and total B cells (CD19^+^) were gated. The expression of mIgD on T and B cells was analyzed by calculating the percentages of CD3^+^IgD^+^, CD3^+^CD4^+^IgD^+^, immature B cells (CD19^+^IgM^+^IgD^-^) and mature B cells (CD19^+^IgD^+^) on a flow cytometer (model FC 500; Beckman Coulter Ltd., USA). The data were analyzed with CXP analysis software (Beckman Coulter Ltd., USA, version 2.0) or FlowJo (Tree Star, Inc., USA, version 7.6.1) software. The above mAbs and mouse IgG1 isotype controls were purchased from BD Pharmingen (San Diego, CA, USA). For analysis, the lymphocyte population was gated based on forward and side scatter.

IgDR expression was detected by treating PBMCs with biotinylated IgD [9]. Human IgD protein (IgD) was purchased from Abcam Inc (Cambridge, MA, UK). Biotinylated IgD was prepared from IgD protein in our laboratory using a protein biotinylation kit from Pierce Biotechnology (Rockford, IL, USA) according to the manufacturer’s instructions. A total of 1 × 10^6^ cells were washed twice with PBS and incubated with 1 μg of biotinylated IgD for 30 min at room temperature. Cells were washed three times with PBS, incubated with streptavidin APC-Cy7 (BD Pharmingen) for 30 min on ice, washed twice, and resuspended in fresh PBS. Data were acquired on a flow cytometer. Cells stained with streptavidin APC-Cy7 alone were used as isotype control.

### The Proliferation of PBMCs Was Analyzed by Cell Counting Kit-8

PBMCs (2.0 × 10^6^ cells/ml) were cultured in RPMI-1640 (HyClone, Carlsbad, CA, USA) with 5% fetal bovine serum (FBS; HyClone, Carlsbad, CA, USA) and were stimulated with different concentration of IgD (0.1, 0.3, 1, 3, 10, or 30 μg/ml, Abcam Inc) for 24, 48, or 72 h in 96-well plates. Control cells were treated with media only.

A cell counting kit-8 (WST-8; Dojindo Laboratories, Kumamoto, Japan) was used to explore the effects of IgD on PBMCs. 10 μl WST-8 solution was added per well after culture. Blank controls included 100 μl RPMI-1640 and 10 μl WST-8 solution in triplicate wells [19]. The cells were incubated for 4 h at 37°C in an atmosphere containing 5% CO_2_, and the absorbance at 450 nm was measured colorimetrically on a microplate reader (model ELx808; BioTek, Winooski, VT, USA), according to the manufacturer’s protocol. Proliferative responses were expressed as a stimulation index (SI), wherein the SI was equal to the absorbance at 450 nm in cells cultured with drug divided by the absorbance in cells cultured with medium alone.

### The Effect of IgD on T and B Subsets Was Measured by Flow Cytometry Analysis

PBMCs from RA patients and healthy controls were treated with different concentration of IgD (1, 3, or 10 μg/ml) or phytohemagglutinin (PHA, 4 μg /ml; Sigma, St. Louis, MO, USA) for 24 h. The supernatant was collected for cytokine assays. Cells were harvested and then incubated with various combinations of monoclonal antibodies (mAbs) for 30 min at room temperature. The percentages of total helper T cells (CD3^+^CD4^+^), total cytotoxic T cells (CD3^+^CD8^+^), activated T cells (CD4^+^CD69^+^, CD4^+^CD154^+^), unactivated T cells (CD4^+^CD62L^+^), immature B cells (CD19^+^IgM^+^IgD^-^), activated B cells (CD19^+^CD23^+^, CD19^+^CD21^+^), mature B cells (CD19^+^IgD^+^) and plasma cells (CD19^-^CD138^+^) were analyzed on a flow cytometer. The above mAbs and mouse IgG1 isotype controls were purchased from BD Pharmingen. For each experiment, control cells were treated with media only.

### Cytokines Assays

The levels of inflammatory cytokines, including IL-1α, IL-1β, TNF-α, IL-6, IL-10, IL-8, IL-4, INF-γ, IL-13, and monocyte chemotactic protein (MCP)-1 in PBMC supernatants were measured by using the Quantibody human inflammation array 1 (RayBiotech, Norcross, USA). Fluorescence intensities were measured with a Genepix 4000B Microarray Scanner (Molecular Devices, Sunnyvale, CA) at a wavelength of 532 nm. The data was processed using the GenePix Pro 7.0 software.

### Statistical Analysis

Statistical analysis was performed by ANOVA, Student’s t-test or nonparametric unpaired Mann-Whitney test by using SPSS 11.5 software (Chicago, IL, USA). ANOVA was used exclusively for multi-group comparisons, and Student’s t-test was only used for independent, two-group comparisons. Data was checked for a normal distribution in order to decide whether to use parametric or non-parametric tests. Median group values (with standard error of the mean) for percentage and absolute numbers of different cell populations were compared in patients and healthy controls using the nonparametric unpaired Mann-Whitney test. Pearson correlation analysis was used to examine the correlation between the parameters. Multivariable linear regression analysis was used to assess the independent contribution of the variables. For proliferation assay, the EC_50_ of stimulating curves were analyzed by GraphPad Prism 4.0. Data were presented as mean ± standard error of mean unless otherwise indicated. *P* < 0.05 was considered statistically significant.

## Results

### Characteristics of the RA Patients and Healthy Controls

The clinical and demographic characteristics of the 54 RA patients and 42 healthy controls in this study are reported in Table 1. Most of the 54 patients were females. The healthy control group had a lower proportion of women compared with the RA group (*P* < 0.001).

**Table 1:** Clinical and demographic characteristics of the RA patients and healthy controls.

Characteristic	Healthy controls	RA
**Number of subjects**	42	54
**Age (years)**	45±8	59±14
**Female/male ratio**	20:22	45:9
**sIgD (μg/ml)**	19.8±6.8	91.93±111.0
**sRANKL (pg/ml)**	44.5±49.5	170.4±139.7
**RF (IU/mL)**	—	118.3±84.2
**ESR (mm/hour)**	—	70±28
**Anti-CCP (RU/mL)**	—	653±553
**CRP (mg/dl)**	—	53.3±58.3

Data are means±standard deviation.

Anti-CCP = anti-cyclic citrullinated peptide; CRP = C-reactive protein; ESR = erythrocyte sedimentation rate; RA = rheumatoid arthritis; RF = rheumatoid factor; sRANKL = soluble receptor activator of nuclear factor-κB ligand.

### IgD Expression in RA Patients Were Higher than Those in Healthy Controls

**Fig 1:**
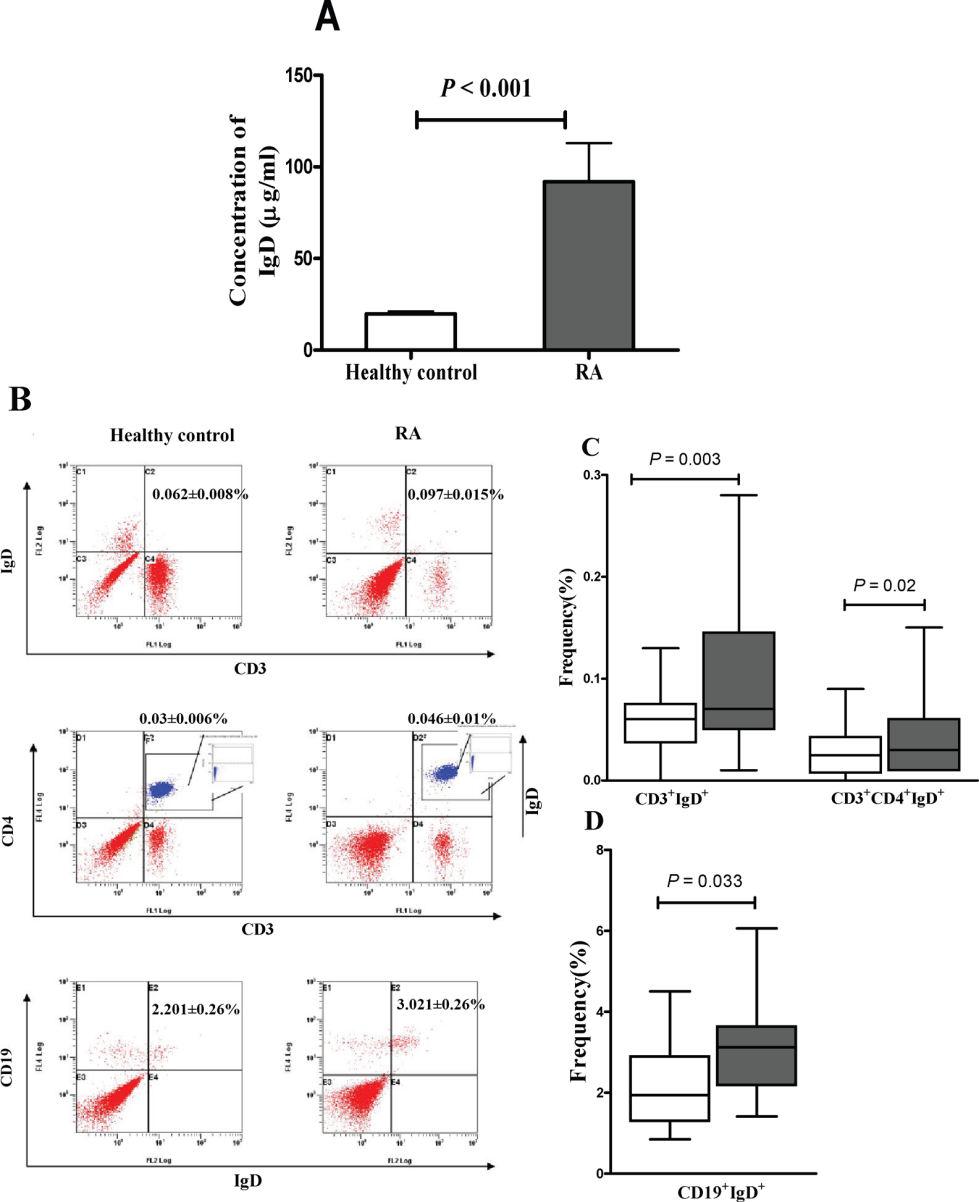
Levels of sIgD and mIgD in RA patients and healthy controls. (A): Serum IgD concentrations were significantly different in RA patients (n = 28) and healthy controls (n = 22) (*P* < 0.001). (B): A dot plot showing the expression of IgD on T cells and B cells. (C) and (D): Box-plots representing the 10th, 25th, 50th (median), 75th and 90th percentiles of the frequencies of the CD3^+^IgD^+^, CD3^+^CD4^+^IgD^+^ and CD19^+^IgD^+^ (each as a percentage of lymphocytes) in the peripheral blood of healthy controls (n = 18, white bars) and RA patients (n = 20, grey bars). *P* < 0.05 was considered statistically significant.

The level of sIgD in serum from RA patients was significantly higher than that in healthy controls (*P* < 0.001) (Table 1 and Fig 1A). Little mIgD was detected on total T cells (CD3^+^) and total helper T cells (CD3^+^CD4^+^), which was consistent with the finding of Chen K et al [6]. The percentage of CD3^+^CD4^+^ was higher in RA patients (27.73±2.35) than healthy controls (18.96±1.33) (*P* = 0.008). Peripheral B cell subsets were altered in patients with RA. The percentages of mature B cells (CD19^+^IgD^+^) was higher in RA patients (3.02±0.26) than healthy controls (2.20±0.26) (*P* = 0.033) (Fig 1B–1D), while that of immature B cells (CD19^+^IgM^+^IgD^-^) was lower in RA patients than healthy controls (0.48±0.10 *vs*. 1.53±0.20, *P* < 0.001[S1 Fig]). In a word, the level of sIgD in serum and the expression of mIgD on B cells were higher in RA patients than that in healthy controls.

### IgD Correlated with sRANKL, RF and CRP

**Fig 2:**
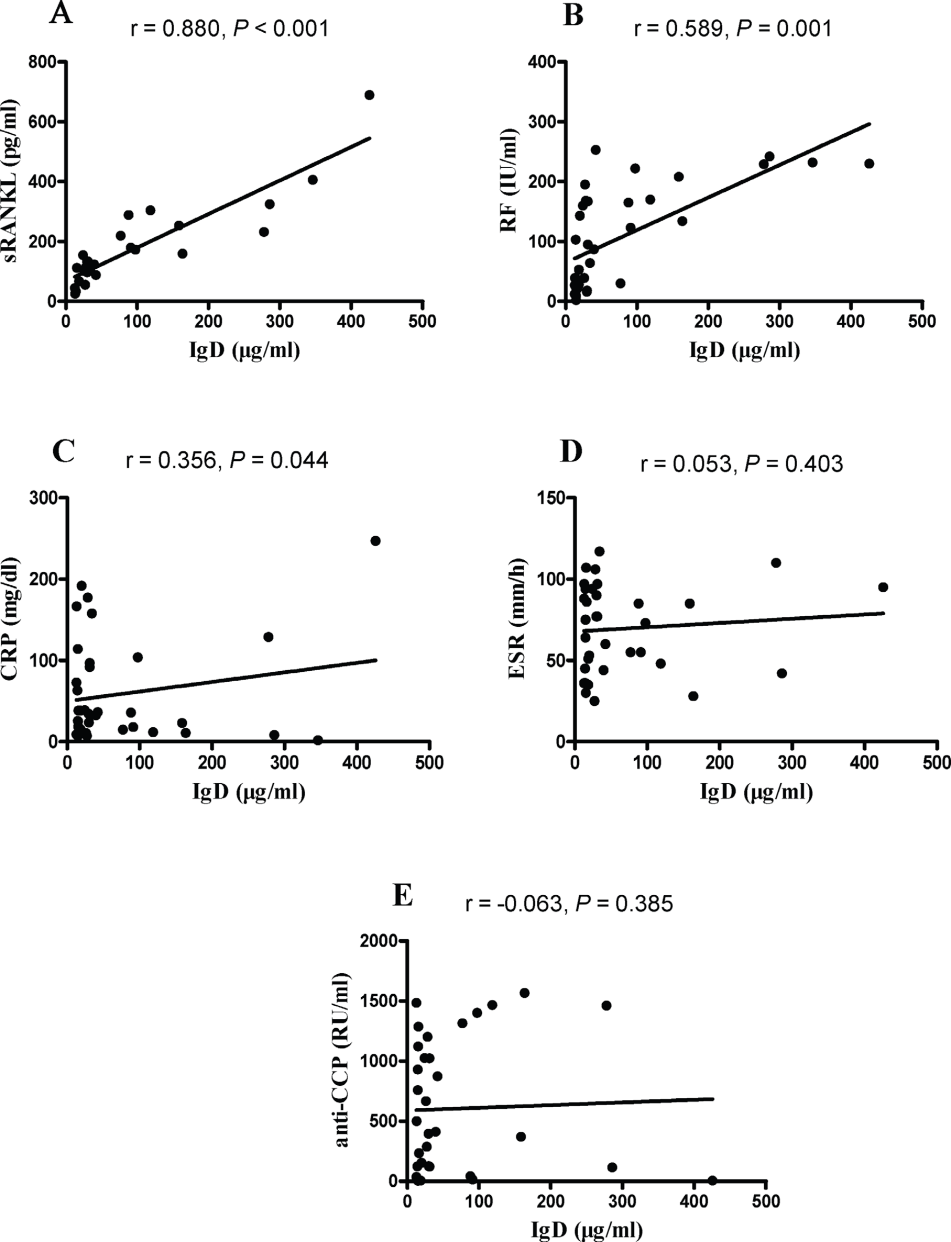
The correlation of the serum IgD levels with biomarkers in RA patients. Pearson correlation analyses were shown for the association of IgD with sRANKL (A), RF (B), CRP (C), ESR (D) and anti-CCP (E).

The sRANKL concentrations in serum were detected and significantly higher in RA patients than those in healthy controls (Table 1, *P* < 0.001). To examine the correlation between the biomarkers of disease and the sIgD level in serum, Pearson correlation analysis was performed between the sRANKL, RF, CRP, ESR, anti-CCP levels of RA and the sIgD concentrations. Interestingly, the sIgD concentrations were positively associated with the sRANKL (r = 0.880, *P* < 0.001), RF (r = 0.589, *P* = 0.001) and CRP (r = 0.356, *P* = 0.044), while negatively with the concentrations of ESR (r = 0.053, *P* = 0.403) and anti-CCP (r = -0.063, *P* = 0.385) (Fig 2).

To determine the independent association between sIgD and biomarkers, multivariable linear regression analysis was performed (Table 2). The results indicated that sIgD concentrations were positively correlated with sRANKL concentrations of all biomarkers (*P* < 0.01). sRANKL concentrations had the strongest correlation with sIgD concentrations in serum.

**Table 2:** Independent associations of the sIgD concentrations with the sRANKL, RF, CRP, ESR and anti-CCP in RA patients.

Variables	Multivariable linear regression analysis	
	Coefficients	*P*-value
**sRANKL**	0.770	<0.001
**RF**	0.174	0.168
**CRP**	0.111	0.436
**ESR**	-0.106	0.437
**Anti-CCP**	0.027	0.795

Multivariable linear regression analysis was used to assess the independent contribution of the variables. sRANKL concentrations had the strongest correlation with sIgD concentrations in serum.

### IgDR Expression in RA Patients Were Higher than Those in Healthy Controls

**Fig 3:**
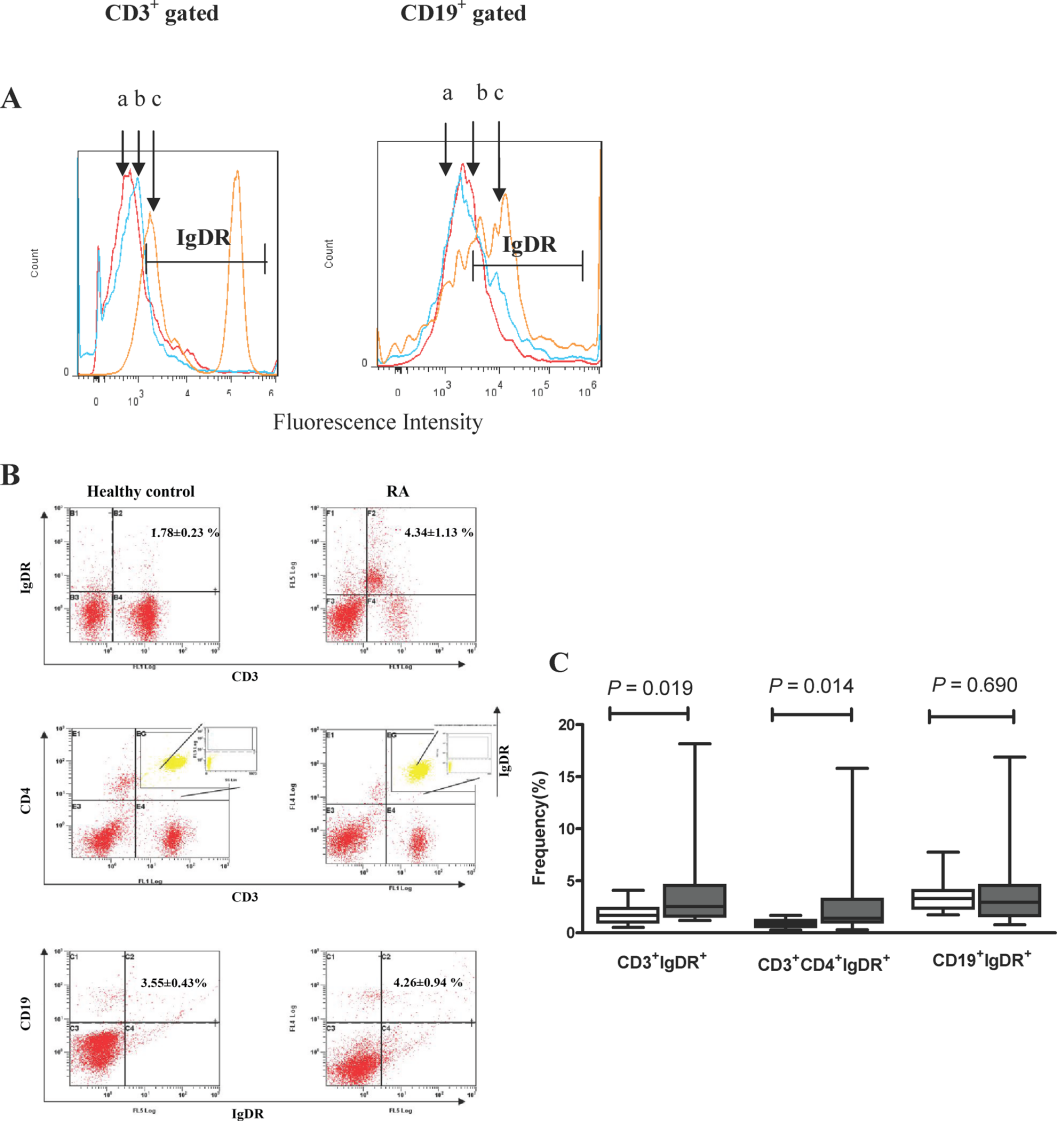
Expression of IgDR on T cells and B cells in RA patients and healthy controls. (A): A histogram showing the expression of IgDR on CD3^+^ T cells and CD19^+^ gated B cells. The fluorescence profile of the stained cells was analyzed by flow cytometry. Red curve (a): isotype; blue curve (b): healthy control; orange curve (c): RA patient. (B): A dot plot showing the expression of IgDR on T cells and B cells. (C): Box-plots representing the 10th, 25th, 50th (median), 75th and 90th percentiles of the frequencies of the CD3^+^IgDR^+^, CD3^+^CD4^+^IgDR^+^ and CD19^+^IgDR^+^(each as a percentage of lymphocytes) in the peripheral blood of healthy controls (n = 15, white bars) and RA patients (n = 19, grey bars). *P* < 0.05 was considered statistically significant.

We established the detection method for IgDR through flow cytometry, and confirmed that IgDRs exist on not only T cells but also B cells. Subsequently, we compared the expression of IgDR on T and B cells between RA and healthy controls. The results showed the expression of IgDR on CD3^+^ T cells and CD4^+^ T cells were significantly higher in RA patients (*P* < 0.05) than those in healthy controls. There is no significant difference of IgDR expression on CD19^+^ B cells between RA patients and healthy controls (Fig 3).

### IgD Enhanced PBMCs Proliferation in RA Patients

**Fig 4:**
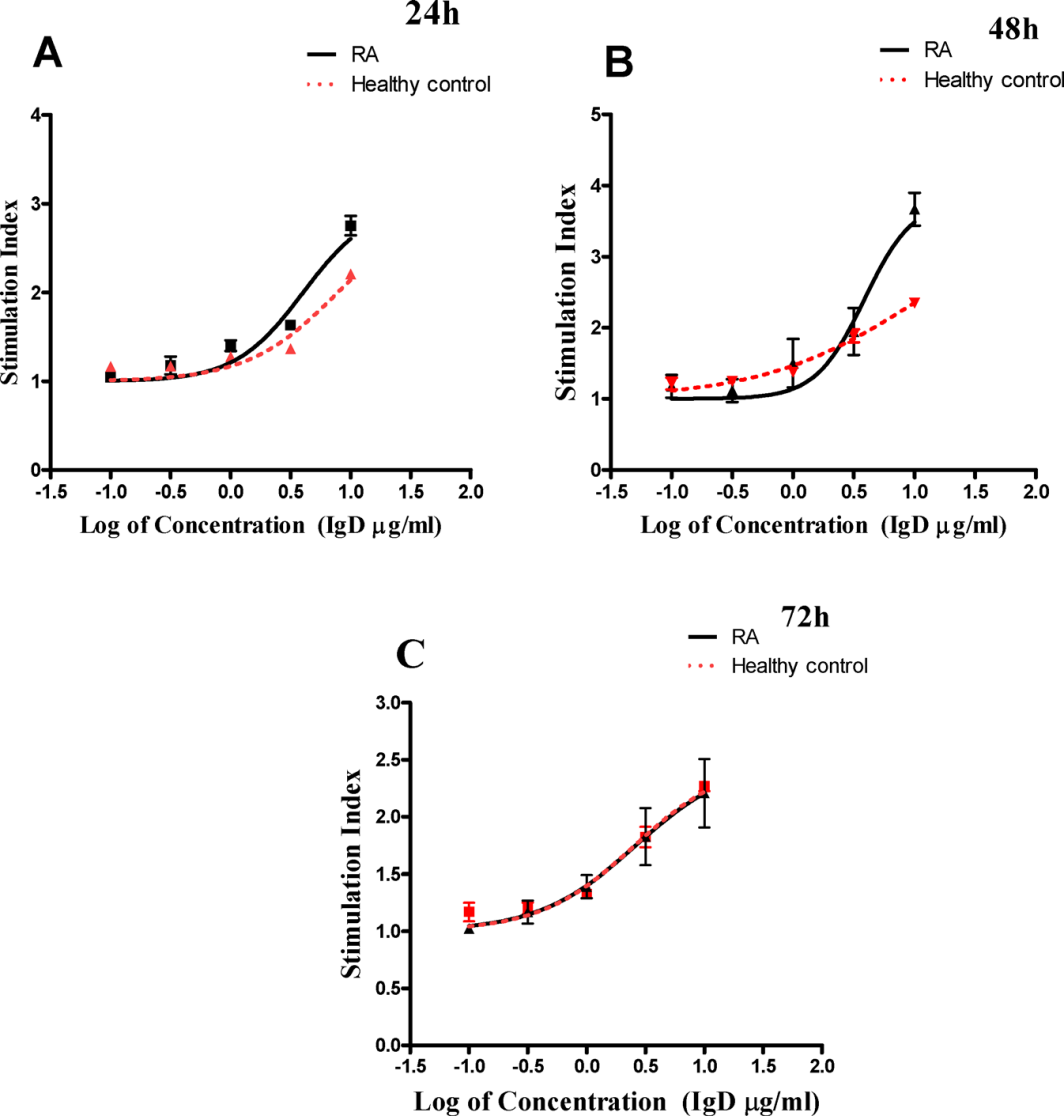
The stimulating effect of IgD on human PBMCs from RA patients and healthy controls. PBMCs from RA patients or healthy controls cultured with 0.1, 0.3, 1, 3, 10 μg/ml of IgD in duration [24h (A), 48h (B), 72h(C)]. Data was expressed as mean±standard error of mean (n = 4), SI = absorbance at 450 nm in the cell cultures with drug divided by absorbance in the culture with medium alone.

PBMCs were cultured with different concentrations of IgD (0.1, 0.3, 1, 3, 10 μg/ml) for 24, 48, and 72 h, and their respective proliferation was measured. As expected, the proliferation of PBMCs increased in response to IgD in both RA patients and healthy controls in a concentration-dependent manner (Fig 4, *P* < 0.05). The stimulating effect of IgD between RA patients and healthy controls was also compared. As shown in Table 3, the response of PBMCs from RA patients to IgD was obviously stronger than that of healthy controls at the same time point. The EC_50_ value was lower in RA groups (EC_50_ = 4.016 μg/ml) than that of healthy control groups (EC_50_ = 7.973 μg/ml) after 24 h of culture. The EC_50_ value was further reduced in RA groups (EC_50_ = 3.869 μg/ml) than that in healthy control groups (EC_50_ = 5.79 μg/ml) after 48 h. There was no obvious difference between the two groups after 72 h. These results suggested that the optimal stimulation time was 24 h, and the optimal stimulation concentration ranges from 1 to 10 μg /ml.

**Table 3:** Comparison of stimulating effect of IgD between RA patients and healthy controls.

Group	RA	Healthy Control	RA	Healthy Control	RA	Healthy Control
**Time(h)**	**24**		**48**		**72**	
**EC**_**50**_ **(μg /ml)**	4.016	7.973	3.869	5.79	2.608	3.426
**95% Confidence Intervals**	2.073~7.778	6.029~10.54	2.220~5.879	3.107~10.79	2.346~2.899	1.763~6.656
**R**^**2**^	0.9393	0.9895	0.9693	0.9530	0.9989	0.9487

### IgD Increased the Production of Inflammatory Cytokines in PBMCs

**Fig 5:**
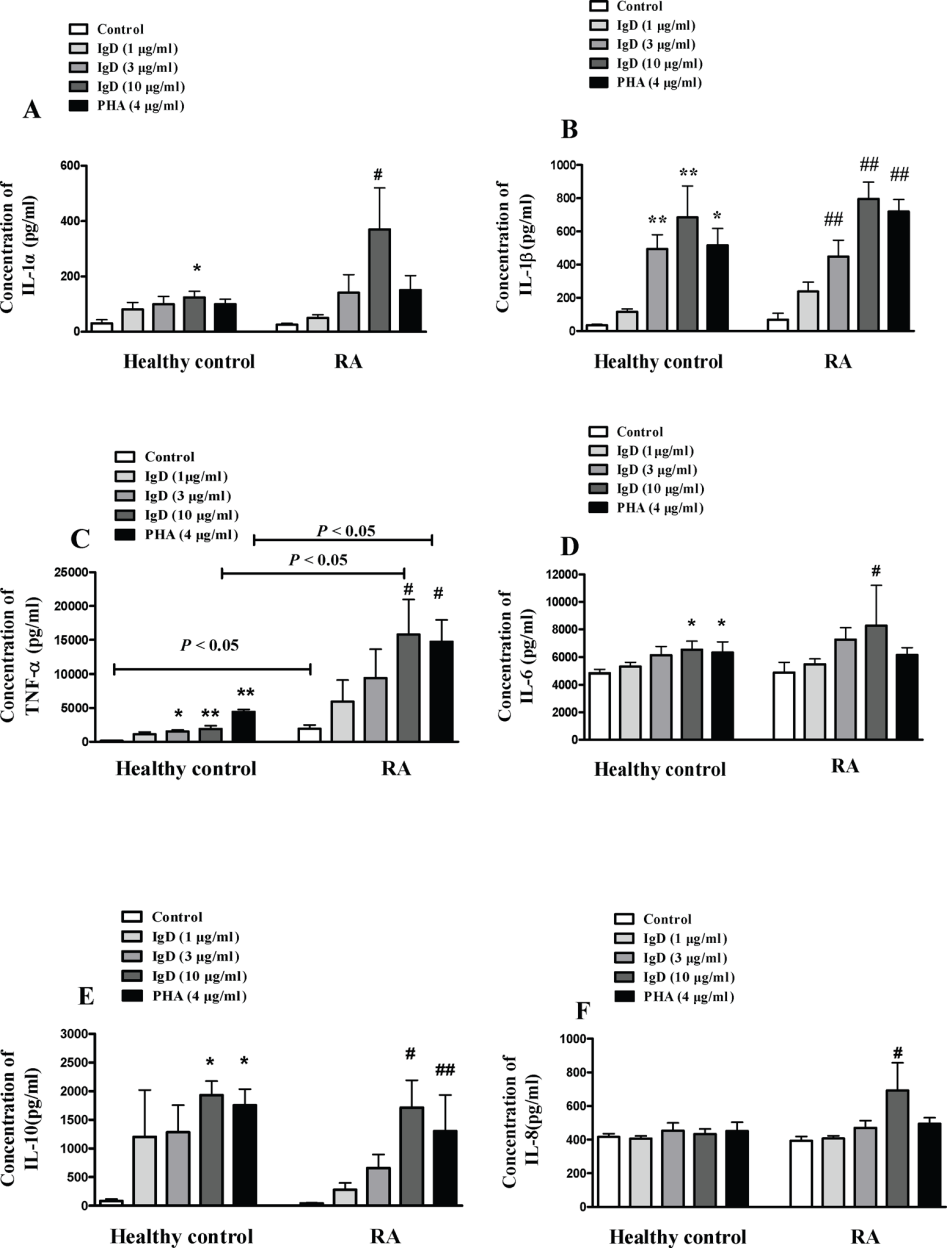
The effect of IgD on levels of inflammatory cytokines in PBMCs culture supernatant. PBMC culture supernatants were collected from RA patients and healthy controls and treated with IgD (1, 3, or 10 μg/ml) or PHA (4 μg/ml) for 24 h. Levels of IL-1α(A), IL-1β(B), TNF-α(C), IL-6(D), IL-10 (E) and IL-8(F) were measured by microarray scanner as described in the Materials and Methods. Data represent the mean ± standard error of the mean (n = 4). *P < 0.05, **P < 0.01 *vs*. the control from healthy controls; ^#^<0.05, ^##^*P* <0.01 *vs*. the control from RA.

To examine whether IgD affects the inflammatory cytokines secretion by PBMCs, inflammatory cytokines were analyzed by the Quantibody human inflammation array 1. Compared with the control group, the concentrations of IL-1α, IL-1β, TNF-α, IL-6, IL-8 and IL-10 were increased significantly in IgD (10 μg/ml) group of RA patients (*P* < 0.05) (Fig 5). There was no obvious difference in the concentrations of IL-4, INF-γ, IL-13 and MCP-1. In healthy controls, the levels of IL-1α, IL-1β, TNF-α, IL-6, and IL-10 were increased significantly in IgD group. The level of TNF-α in PBMCs supernatants of RA patients was significantly higher than that of healthy controls.

### IgD Enhanced Activated T Cell and Decreased Unactivated T Cell Subsets

**Fig 6:**
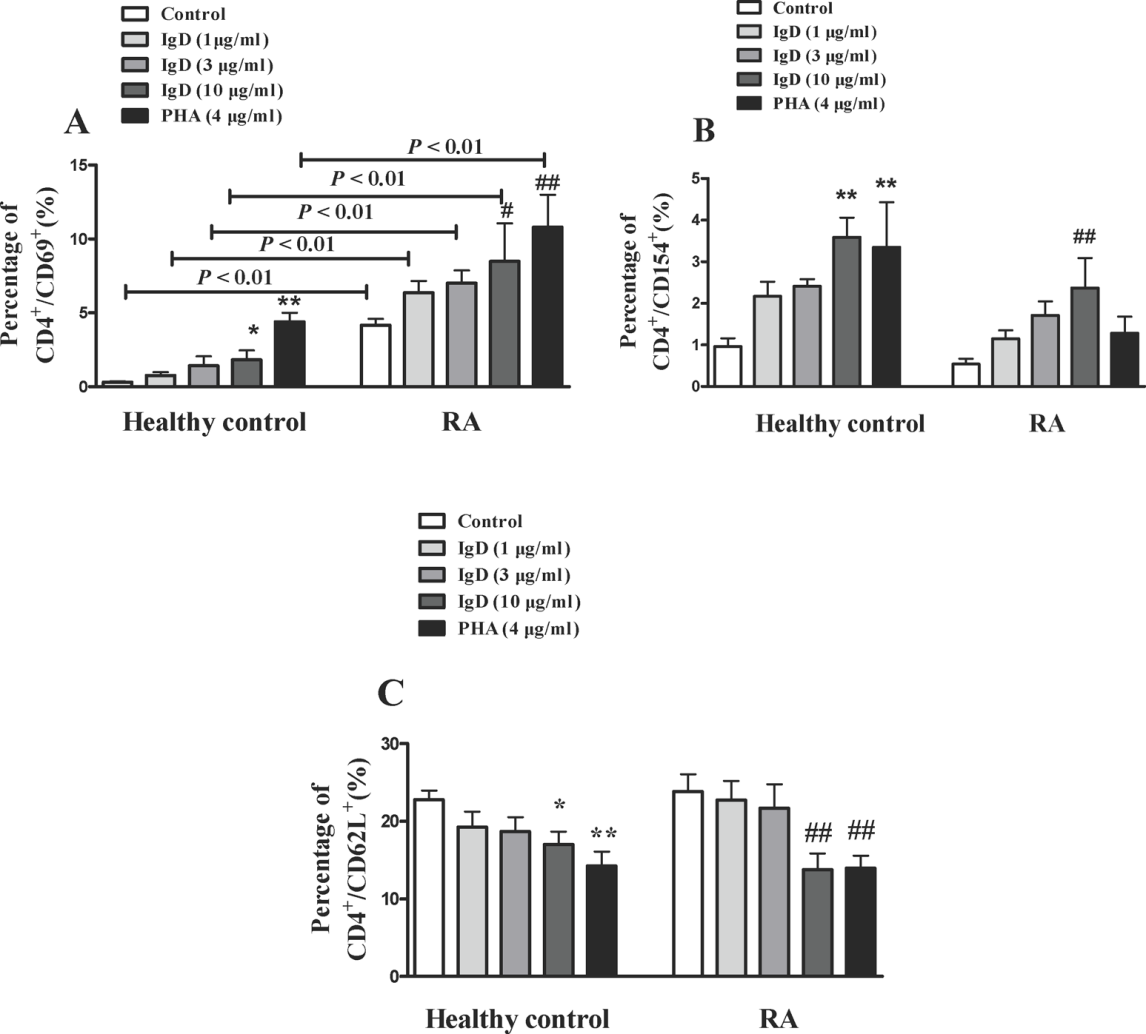
The effects of IgD on subsets of T cells in PBMCs from RA patients and healthy controls. Flow cytometry analysis was used to analyze the effects of IgD (1, 3, or 10 μg/ml) or PHA (4 μg/ml) on activated T cells (CD4^+^CD69^+^, CD4^+^CD154^+^) and unactivated T cells (CD4^+^CD62L^+^). Data are expressed as the mean ± standard error of the mean (n = 8). *P < 0.05, **P < 0.01 *vs*. the control from healthy controls; ^#^*P* <0.05, ^##^*P* <0.01 *vs*. the control from RA.

The effects of IgD on T cell activation were analyzed (Fig 6). The results showed that the percentages of activated T cells (CD4^+^CD69^+^, CD4^+^CD154^+^; *P* < 0.05) were increased and the percentage of unactivated T cells (CD4^+^CD62L^+^; *P* < 0.05) was decreased after co-cultured with IgD (10 μg/ml) or PHA (4 μg/ml) for 24h in RA patients. IgD had no significant effect on total helper T cells (CD3^+^CD4^+^) or total cytotoxic T cells (CD3^+^CD8^+^). In healthy controls, the effect of IgD on T cell subsets was similar with the results observed in RA patients. Interestingly it was observed that, the percentage of activated T cells (CD4^+^CD69^+^) in RA patients was higher than that in healthy controls.

### IgD Enhanced Activated B Cell Subsets in RA Patients

**Fig 7:**
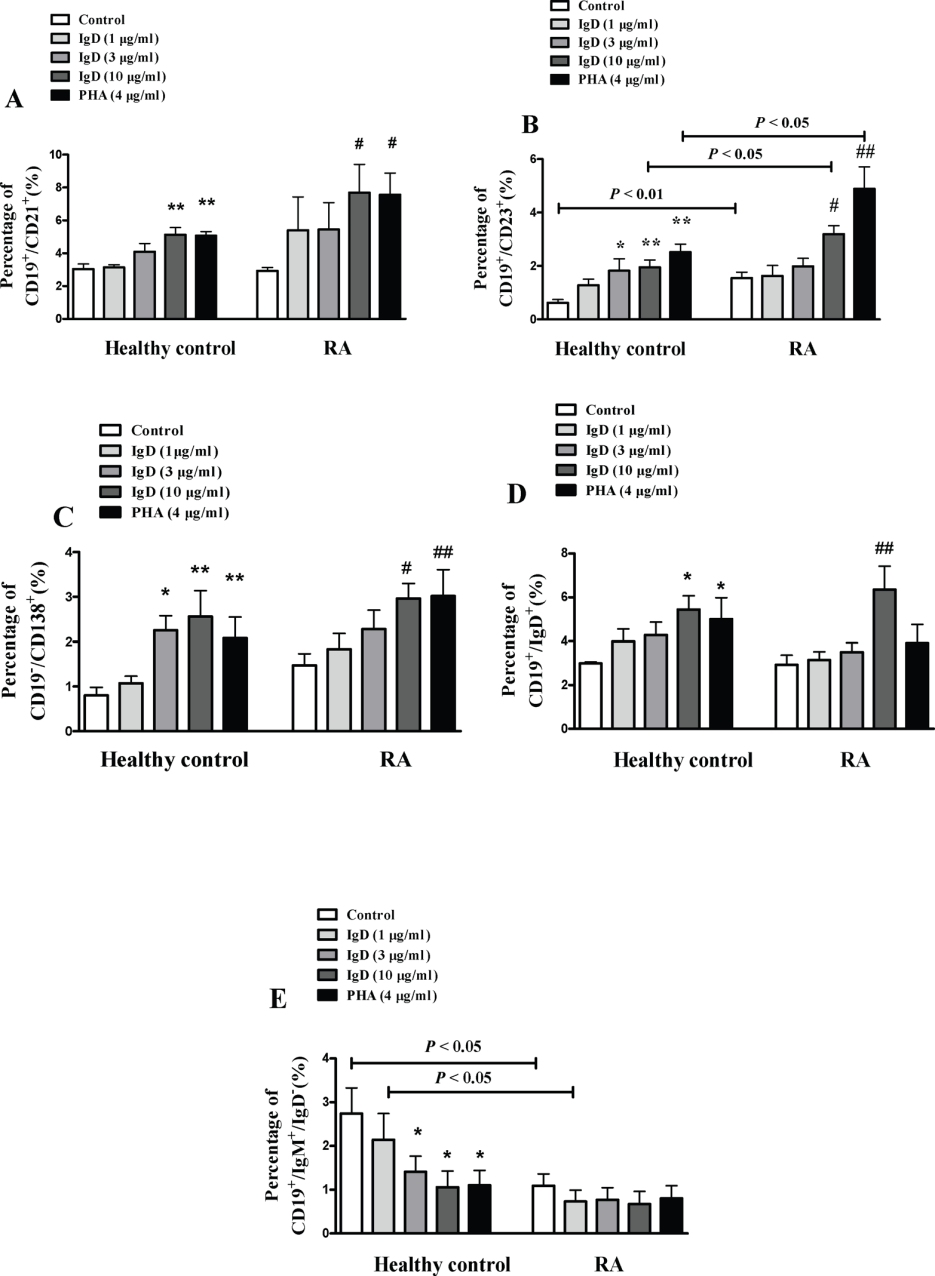
The effects of IgD on subsets of B cells in PBMCs from RA patients and healthy controls. Flow cytometry analysis was used to analyze the effects of IgD (1, 3, or 10 μg/ml) or PHA (4 μg/ml) on the percentages of activated B cells (CD19^+^CD23^+^, CD19^+^CD21^+^), mature B cells (CD19^+^IgD^+^), plasma cells (CD19^-^CD138^+^), and immature B cells (CD19^+^IgM^+^IgD^-^). Data are expressed as the mean ± standard error of the mean (n = 8). *P < 0.05, **P < 0.01 *vs*. the control from healthy controls; ^#^*P* <0.05, ^##^*P* <0.01 *vs*. the control from RA.

It was found that IgDR existed on not only T cells but also on B cells, the effects of IgD on B cell activation were analyzed in the meanwhile (Fig 7). The results showed that the percentages of activated B cells (CD19^+^CD23^+^, CD19^+^CD21^+^), mature B cells (CD19^+^IgD^+^), and plasma cells (CD19^-^CD138^+^; *P* < 0.05) were increased and the percentage of immature B cells (CD19^+^IgM^+^IgD^-^) was slightly decreased (though not significantly) when co-cultured with IgD (10 μg/ml) or PHA (4 μg/ml) in RA patients. IgD had no significant effect on total B cells (CD19^+^). Consistent with healthy controls, the effect of IgD on B cell subsets was similar with the results observed in RA patients. Interestingly, the percentage of CD19^+^CD23^+^ was higher, and that of CD19^+^IgM^+^IgD^-^ was lower in RA patients than in healthy controls. Also we observed that the percentage of immature B cells in healthy controls when co-cultured with IgD decreased significantly to a level which was similar to RA patients.

### IgD Promoted IgDR Expression in PBMCs of RA Patients

**Fig 8:**
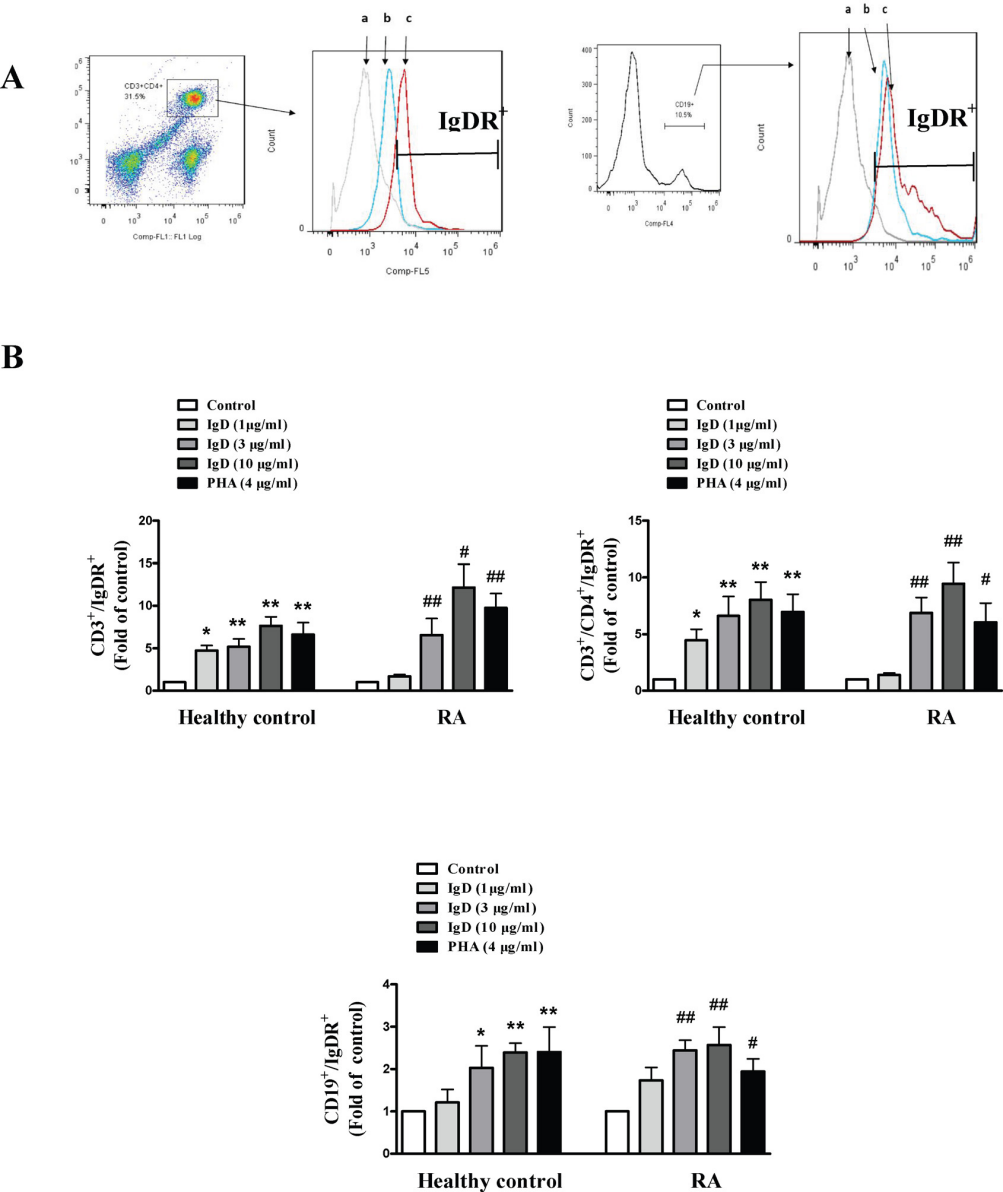
The effects of IgD on IgDR expression in PBMCs from RA patients and healthy controls. (A): A histogram showing the expression of IgDR on CD3^+^CD4^+^-gated T cells and CD19^+^-gated B cells. The fluorescence profile of the stained cells was analyzed by flow cytometry. Curve a: isotype control; curve b: control group; curve c: IgD (10 μg/ml) group. (B): Effects of IgD treatment (1, 3, or 10 μg/ml) on the percentages of CD3^+^IgDR^+^ T cells, CD3^+^CD4^+^IgDR^+^ T and CD19^+^IgDR^+^ B cells. Data are expressed as the mean ± standard error of the mean (n = 6). *P < 0.05, **P < 0.01 *vs*. the control from healthy controls; ^#^*P* <0.05, ^##^*P* <0.01 *vs*. the control from RA.

The expression of IgDR on T and B cells stimulated by IgD after 24 h were analyzed in both RA patients and healthy controls (Fig 8). IgDR was presented on a small percentage of T cells (<5%) and B cells (<5%) in PBMCs from both RA patients and healthy controls. The percentage of CD3^+^IgDR^+^ T cells, CD3^+^CD4^+^IgDR^+^ T and CD19^+^IgDR^+^ B cells showed a significant increase (*P* < 0.05) in PBMCs from both RA patients and healthy controls, which was caused by IgD. In particular, the percentage of CD3^+^IgDR^+^ T cells and CD19^+^IgDR^+^ B cells were increased by nearly 12 folds and 2.5 folds by IgD in RA patients respectively. In both RA patients and healthy controls, the expression of IgDR on CD3^+^ T cells, CD3^+^CD4^+^ T cells, and CD19^+^ B cells were also increased by PHA (*P* < 0.05).

## Discussion

RA is a systemic autoimmune disease characterized by synovial membrane hyperplasia and infiltration of inflammatory cells, including activated T and B cells [20]. Lymphoid neogenesis develops with the formation of ectopic germinal centers in approximately 20% of patients [21].

The sIgD, secreted by B cells, was once considered as nonfunctional, because of its low level in serum. However, some reports have shown showing that sIgD is often elevated in RA [12]. High levels of cell necrosis and protein-like sediments have also been observed in the liver, spleen, and kidney of transgenic mice expressing high levels of sIgD [14].

Consistent with previous studies, we found that sIgD level in the serum of RA patients was significantly higher than that in healthy controls. Importantly, we found that sRANKL, RF and CRP, which are the serum biomarkers to assess disease activity and predict disease progression for RA patients, were significantly correlated with the production of sIgD. CRP plays an important role in bone destruction in RA through induction of sRANKL expression and direct differentiation of osteoclast [22]. This finding indicated that sIgD in circulation may be a biomarker for the diagnosis of RA, hence, excessive activation and high expression of IgD might be targets for elimination [23, 24].

Our data confirmed that not only sIgD in serum was elevated in RA, but also the expressions of mIgD and IgDR on T cells and B cells were elevated. In this study, mature B cells (CD19^+^IgD^+^) were observed to be higher in RA patients than that in healthy controls. Moreover, the expression level of IgDR on T cell subsets was higher in RA patients than observed in control populations. The presence of IgDR on T cells was proposed as early as 1985 [8], however, in contrast to IgG and IgE receptors, the structural and functional characteristics of the IgD receptor are still unclear, probably because the gene encoding the IgD receptor has not yet been cloned. Generally, the expression of IgDR was detected by indirect methods [8, 25]. For example, in previous studies, IgDR was measured via sheep erythrocytes coated with IgD as an indicator for enumerating the frequency of IgD rosette-forming cells [6].

In this study, biotinylated IgD and flow cytometry analysis was established to detect more accurately the expression of IgDR on human T and B cells (Fig 1A and S2 Fig). Previous reports suggested the existence of IgDR on mice and human T cells [8–10]. Here, IgDR expressing on both T and B cells was observed, which was consistent with the previous finding that IgDR was expressed on both T cells and non-T cells [26]. Our results further suggested that more active T and B cells were observed in RA patients. The abnormal level of IgD and IgDR in RA may contribute to the RA progression.

In order to investigate whether excessive sIgD participate in the pathogenesis in RA, we analyzed the effects of sIgD on PBMCs proliferation, inflammatory cytokine production, T and B cell activation and IgDR expression in RA patients and healthy controls *in vitro*. The results showed that IgD could enhance the proliferation of human PBMCs *in vitro*, which was more sensitive in RA than in healthy controls. Furthermore, the optimal stimulation time for IgD was 24 h, while that for PHA was 48–72 h (data not shown). The results suggest that IgD is a more effective stimulant for PBMCs. At the same time, IgD could significantly increase the levels of inflammatory cytokines including IL-1α, IL-1β, TNF-α, IL-6 and IL-8 secreted by PBMCs in RA patients. The role of these cytokines in the development of autoimmune disorders is well established [27]. IL-1 and IL-6 are key inflammatory cytokines that regulate tissue destruction and joint inflammation [28]. IL-6 stimulates the differentiation and activation of T and B cells and enhances the production of Igs and stimulates T cells to produce sRANKL [29]. TNF-α plays an important role in RA through influencing earlier stages of the autoimmune process, such as T cell activation and differentiation [30]. In addition, the production of IL-1, IL-6 and TNF-α were released from activated T cells in RA patients, and are considered to be important participants in the pathophysiology of RA [31]. These cytokines were increased after treatment with IgD.

T cell activation is usually along with increased expression of T cell activation markers, such as CD69 and CD154 (CD40L) [32].CD69 is a co-stimulatory signal for T cell proliferation, activation and differentiation [33].CD154 takes part in the development of tumors and inflammation [34]. B cells have a longstanding role in the pathogenesis of RA. CD21 (CR2) plays a role in the proliferation and transformation of B cells, as well as the production of antibodies. Additionally, CD21 promotes B cell responses and promote innate and acquired immunity. Both CD21 and CD23 were used as markers of activated B cells [35]. The percentages of CD4^+^CD69^+^, CD4^+^CD154^+^, CD19^+^CD23^+^, CD19^+^CD21^+^, CD19^+^IgD^+^, and CD19^-^CD138^+^ were increased and the percentages of CD4^+^CD62L^+^ were decreased in RA patients after treatment of IgD, which suggest that IgD promote the activation and maturation of T cells and B cells, and enhanced the activation of plasma cells.

Injection with oligomeric IgD can induce a rapid increase in CD4^+^IgDR^+^ T cells in mice [10]. In mice, crosslinking of IgDR might induce signal transduction through one or more protein tyrosine kinase (PTK) activation pathways [9] or through regulation of a Ca^2+^-dependent protein kinase together with a change in cAMP levels, leading to upregulation of IgDR and T cell activation [11]. In this study, we found that IgD increased the percentage of IgDR on B cells following 24h stimulation as well as on T cells, suggesting that T and B cells were stimulated via IgDR cross-linking by human IgD. Furthermore, the total protein expression of IgDR and phosphorylation of Lck were enhanced by IgD in mouse T cells [9]. It is known that activation of the non-receptor tyrosine kinase Lck is a required step in T cell activation [36]. Our findings illustrate that sIgD could activate T cells, promote the proliferation and secretion of inflammatory cytokines, possibly by enhancing the phosphorylation of Lck, while, activating B cells through increasing CD154^+^ T cells to improve T and B cells interaction. Further elucidation of IgDR and its signaling pathway in the future would be needed to provide a clearer understanding of molecular mechanisms that regulate T and B cell activation.

## Conclusions

This study provides firstly, the evidence that in RA patients, excessive IgD enhanced the proliferation of PBMCs and increased the secretion of inflammatory cytokines which may be caused by the activation of T and B cells via stimulation of IgDR. Meanwhile, the level of sRANKL, RF and CRP in serum increased. A positive feedback loop involving sIgD and IgDR may contribute to the pathogenesis of RA. Because of the restriction of sample size, a large sample-size investigation between RA patients and healthy controls is warranted. Future longitudinal studies will also be helpful to address the relationship between excessive IgD and the pathogenesis of RA, and the potential role of IgD therapies to restore normal immune responses in RA. Finally, this study presents IgD as a potential novel therapeutic target for the management of arthritis as well as other autoimmune diseases that involve both T and B cells.

## Supporting Information

S1 Fig The variation of CD19^+^IgM^+^IgD^-^B cells measured by flow cytometry in RA patients (n = 18) and healthy controls (n = 16). (DOCX)

S2 Fig CD3^+^ T cells and CD19^+^ B cells constitutively expressed IgDR. (DOCX)
