# Supplementary material for: The Elevated Secreted Immunoglobulin D Enhanced the Activation of Peripheral Blood Mononuclear Cells in Rheumatoid Arthritis
Source: PLoS One. 2016 Jan 27;11(1):e0147788. doi: 10.1371/journal.pone.0147788 (PMC4729477; doi:10.1371/journal.pone.0147788)
Supplement: S1 Fig — (DOCX) [file pone.0147788.s001.docx]

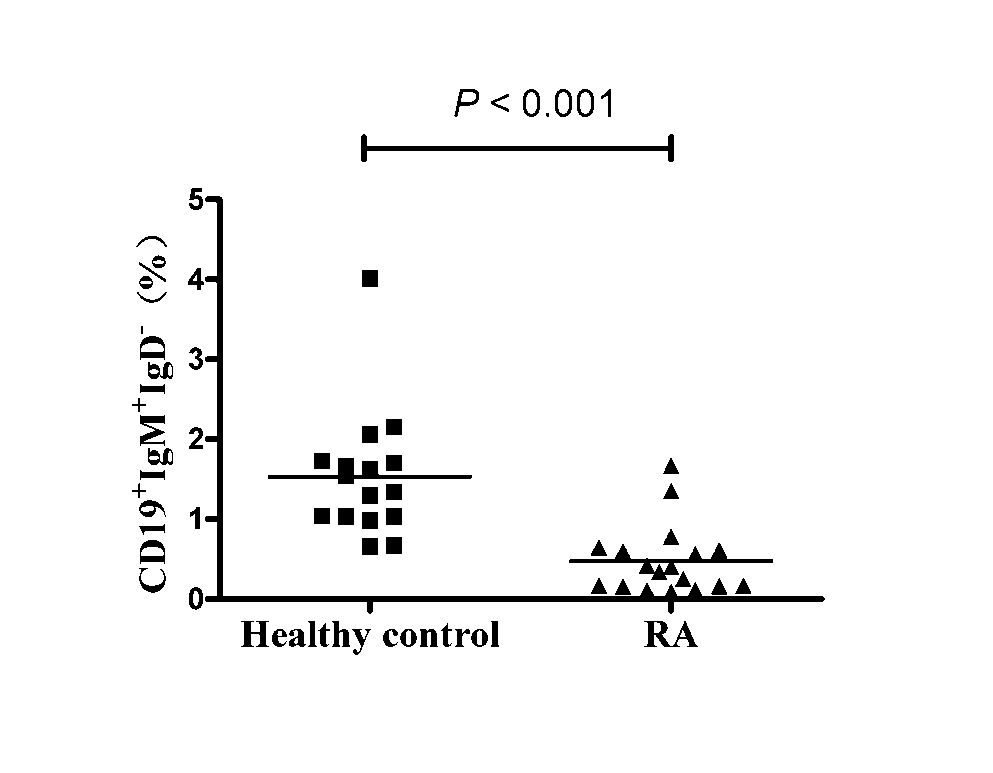


**S1 Fig: The variation of CD19^+^IgM^+^IgD^-^B cells measured by flow cytometry in RA patients (n=18) and healthy controls (n=16).** In RA patients, the percentage of CD19^+^IgM^+^IgD^-^ in peripheral blood was significantly lower than that in healthy controls (*P* < 0.001).
