# Supplementary material for: The Elevated Secreted Immunoglobulin D Enhanced the Activation of Peripheral Blood Mononuclear Cells in Rheumatoid Arthritis
Source: PLoS One. 2016 Jan 27;11(1):e0147788. doi: 10.1371/journal.pone.0147788 (PMC4729477; doi:10.1371/journal.pone.0147788)
Supplement: S2 Fig — (DOCX) [file pone.0147788.s002.docx]

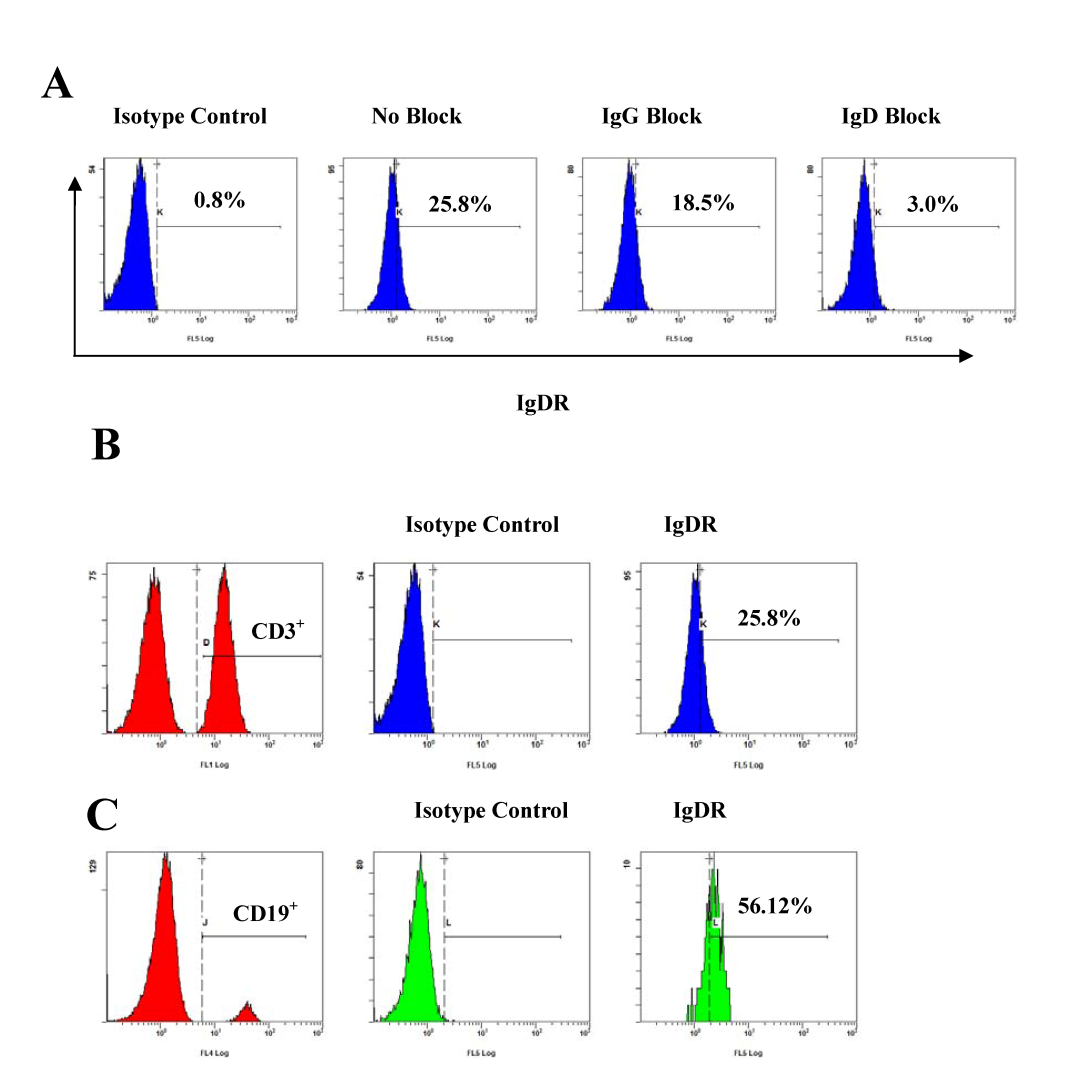


**S2 Fig: CD3^+^ T cells and CD19^+^ B cells constitutively expressed IgDR.** Human PBMCs were analyzed by flow cytometry with 1 µg of biotinylated IgD followed by streptavidin APC-Cy7. 25.8% of CD3^+^ T cells were positively stained with biotinylated IgD. Staining with biotinylated IgD was blocked to 3.0% when cells were simultaneously exposed to unconjugated IgD during staining. In contrast, simultaneous incubation of CD3^+^ T cells with IgG and biotinylated IgD reduced the percentage of cells stained to 18.5 %. Additionally, CD19^+^ B cells constitutively expressed IgDR; 56.12 % of CD19^+^ B cells were positively stained with biotinylated IgD. (A): The specificity of IgDR binding to IgD was confirmed by blocking with unconjugated human IgG (100 µg/ml) or human IgD (100 µg/ml). Cells stained with streptavidin APC-Cy7 alone were used as isotype control. The percentages of IgDR in CD3^+^ gated T cells were determined as follows: isotype control, 0.8 %; no block, 25.8 %; IgG block, 18.5 %; and IgD block, 3.0 %. (B): Percentages of IgDR in CD3^+^ gated T cells. (C): Percentages of IgDR in CD19^+^ gated B cells.
